# Numerical Analysis of Radiation Effects on Fiber Optic Sensors

**DOI:** 10.3390/s21124111

**Published:** 2021-06-15

**Authors:** Sohel Rana, Harish Subbaraman, Austin Fleming, Nirmala Kandadai

**Affiliations:** 1 Department of Electrical and Computer Engineering, Boise State University, Boise, ID 83725, USA; sohelrana@u.boisestate.edu (S.R.); harishsubbaraman@boisestate.edu (H.S.); 2 Idaho National Laboratory, 1955 N Fremont Avenue, Idaho Falls, ID 83415, USA; austin.fleming@inl.gov

**Keywords:** fiber Bragg grating (FBG), long-period grating (LPG), Fabry-Perot (F-P), radiation effects, optical fiber sensors

## Abstract

Optical fiber sensors (OFS) are a potential candidate for monitoring physical parameters in nuclear environments. However, under an irradiation field the optical response of the OFS is modified via three primary mechanisms: (i) radiation-induced attenuation (RIA), (ii) radiation-induced emission (RIE), and (iii) radiation-induced compaction (RIC). For resonance-based sensors, RIC plays a significant role in modifying their performance characteristics. In this paper, we numerically investigate independently the effects of RIC and RIA on three types of OFS widely considered for radiation environments: fiber Bragg grating (FBG), long-period grating (LPG), and Fabry-Perot (F-P) sensors. In our RIC modeling, experimentally calculated refractive index (RI) changes due to low-dose radiation are extrapolated using a power law to calculate density changes at high doses. The changes in RI and length are subsequently calculated using the Lorentz–Lorenz relation and an established empirical equation, respectively. The effects of both the change in the RI and length contraction on OFS are modeled for both low and high doses using FIMMWAVE, a commercially available vectorial mode solver. An in-depth understanding of how radiation affects OFS may reveal various potential OFS applications in several types of radiation environments, such as nuclear reactors or in space.

## 1. Introduction

Nuclear power is a potential clean energy solution to the world’s ever-increasing energy demands [1,2]. Power-producing reactors require little in-core instrumentation; however, accurate in-pile measurements are required to qualify new nuclear fuels or extend the burnup of existing fuel designs. Typical commercial electronic sensors often fail under radiation environments such as those encountered in nuclear reactors, so an alternative approach is needed to monitor physical parameters such as temperature, strain, and pressure [3,4,5,6,7]. Due to their high immunity to electromagnetic interference, optical-fiber-based technologies are considered potential candidates for in-pile nuclear environments [3,6]. However, an in-depth understanding of how radiation affects optical fibers and optical fiber sensors (OFS) is required to ensure their reliability and survivability. Early work has shown that three types of resonance-based OFS—fiber Bragg grating (FBG), long-period grating (LPG), and Fabry-Perot (F-P)—have been widely tested in nuclear environments [8,9,10,11,12,13,14,15]. Studies show that the survivability and performance of OFS in nuclear environments largely depends on the chemical composition of the fibers, the nature of the irradiation, the manufacturing conditions, and the wavelength of light used [3,16]. However, the viability of OFS under a known radiation field is threatened due to radiation-induced modification of their fiber optic properties via three mechanisms: (i) radiation-induced attenuation (RIA), which increases the linear absorption due to radiation-induced defects; (ii) radiation-induced emission (RIE), which adds noise to the useful signal through emitted radiation; and (iii) radiation-induced compaction (RIC), which alters the density and hence the refractive index (RI) of the optical fibers via a knock-on process [3,6].

Radiation-induced attenuation (RIA) increases the linear absorption of the fiber material due to radiation-induced defects [3]. RIA reduces the amplitude of the spectral response and is caused by radiation-induced defects in silica material, resulting in modification of the fiber absorption band. These defects are primarily a function of fiber composition, and a significant amount of work has been performed to mitigate such defects, including studying fiber composition and irradiation conditions, choosing an appropriate wavelength of light, and optimizing manufacturing conditions. [3,5,6,7,8,9,17]. The fiber darkening associated with RIA modifies the complex RI of the material in accordance with a Kramer–Kronig relation [18], and this change in RI is uniform throughout the sensor. The change in RI modifies the numerical aperture of the fiber, again showing up as attenuation. Hence, one can say that RIA attenuates the signal received, without affecting the frequency response of the sensor.

Radiation-induced compaction (RIC) occurs when radiation modifies the density of a fiber, leading to compaction in the overall fiber structure. It has been reported that, under fast neutron irradiation, silica tends to undergo roughly 3% density increase that results in changing the RI by 0.5% [19,20]. This density change varies the volume if the compaction is homogeneous [19,20]. The change in density due to RIC results in a significant spectral shift in the response of resonance-based sensors [16]. In other words, RIC shifts the resonant wavelength position and changes the magnitude of the propagating signal, thus corrupting the signal response. Therefore, while RIA modifies the signal strength, RIC modifies both the intensity and wavelength shift response. Subsequently, RIC is the primary cause of data error that occurs in the performance of resonance-based sensors.

Radiation-induced emission (RIE) adds noise to the useful signal through emitted radiation [3] from defects excited by radiation or Cherenkov emissions [8]. RIE is generated when pre-existing or radiation-induced point defects are excited by incoming particles, causing a parasitic signal (radiation-induced luminescence or Cherenkov emissions) [21]. This parasitic signal superimposes onto the transmitted data and decreases the signal-to-noise ratio of the fiber system. At room temperature, thermal bleaching of radiation-induced point defects during or after irradiation partially recovers the transmission. It was suggested that thermal annealing (close to room temperature) only untraps the charge carriers from the defect sites, but defect sites remain unchanged and filled up again during the irradiation [22]. For applications in which the sensor operates in the infrared domain, the effect of RIE is neglected. RIE is generally considered in the ultra-violet (UV)-visible wavelength spectrum [3]. As a result, we did not analyze the effects of RIE on these sensors at the infrared wavelength.

In this paper, we numerically analyze the macroscopic effects of RIC on three resonance-based types of sensors: FBG, LPG, and F-P. We started our simulation by looking the effects of variation of RI on these sensors caused by gamma radiation. As the radiation causes change in the structure of optical sensors, which results in macroscopic changes, for example, RI change in the fiber material, the simulation indirectly also analyzes the radiation effects on the optical structure. We obtained the values for radiation-induced RI changes in optical fibers, as experimentally determined by Kher et al. [18]. Based on the radiation-induced RI values in [18], we used the Lorentz–Lorenz relation to calculate the corresponding density and length change in the optical fiber and then studied its effects on sensor response. We used established empirical models to extrapolate the RI variation at higher doses to predict the response of these sensors at such doses. Finally, we hypothesized the usability of each sensor in various radiation environments. We also included our preliminary results on how RIA affects the performance of these sensors. The paper is divided up as follows: Section 2 describes the modeling of FBG, LPG, and F-P sensors; Section 3 analyzes the effects of RIC on these three types of sensors at low doses; Section 4 investigates how RIC affects these three types of sensors at high doses; Section 5 discusses the effects of RIA on these three types of sensors; and Section 6 offers concluding remarks based on our observations.

## 2. Setting up the OFS Models

To numerically investigate how radiation affects optical performance, we modeled three types of resonance-based sensors (FBG, LPG, and F-P) using a robust and fully vectorial mode solver (FIMMWAVE from PhotonDesign) [23]. This simulation software can solve a large variety of waveguides made of almost any material and any geometry as it supports a rich number of complementary algorithms. FIMMPROP, the integrated part of FIMMWAVE, is a tool for simulating propagation in optical waveguides. For modelling grating based sensors, FIMMPROP uses either Eigenmode Expansion (EME) method or rigorous coupled mode theory (RCMT). SMF-28 optical fiber parameters used for simulation purposes throughout the paper are: cladding RI = 1.444 [24], core diameter = 8.2 µm, cladding diameter = 125 µm, mode field diameter = 10.4 ± 0.5 µm, and numerical aperture = 0.14. The following sections describe the base model for the three types of OFS in this paper.

### 2.1. Fiber Bragg Grating (FBG)

**Figure 1:**
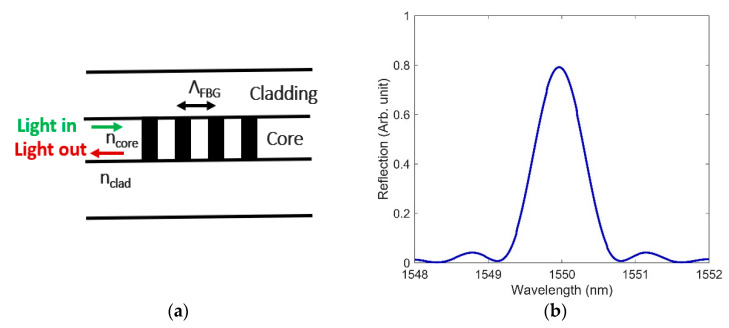
(**a**) FBG schematic, where $n_{core}$,$\;n_{clad}$, and $\Lambda_{FBG}$ are the RIs of the core, cladding, and grating period, respectively; (**b**) the reflection spectrum of the designed FBG for $\Lambda_{FBG}$= 0.5353 µm and grating strength ($\Delta n)=10^{-4}$.

FBG inscription in the core of the fiber using femtosecond (fs) laser radiation and changing the refractive index of the core have found potential applications in nuclear environments, as reported in [9,10,17,25,26]. An FBG is created by generating a periodic RI modulation in the fiber core. In an FBG, the fundamental guided mode couples to the counterpropagating guided mode when the following phase-matching condition is satisfied [27]:

(1) $$\lambda_{B}=2n_{eff}\Lambda_{FBG}$$

where $\lambda_{B}$ is the Bragg wavelength, $n_{eff}\;$ is the effective RI of the core, and $\Lambda_{FBG}$ is the grating period. The Bragg wavelength is very sensitive to any external changes in physical parameters such as temperature and pressure. A schematic of the FBG is shown in Figure 1a, where $n_{core}$ and $n_{clad}$ are the RIs of the core and cladding, respectively. To design an FBG in FIMMWAVE, we used a grating period of 0.5353 µm to obtain a Bragg peak near 1550 nm. The effective RI we obtained for the designed FBG is 1.447714. The reflection spectrum of the designed FBG is shown in Figure 1b with a distinct peak at 1550 nm observed.

### 2.2. Long-Period Grating (LPG)

**Figure 2:**
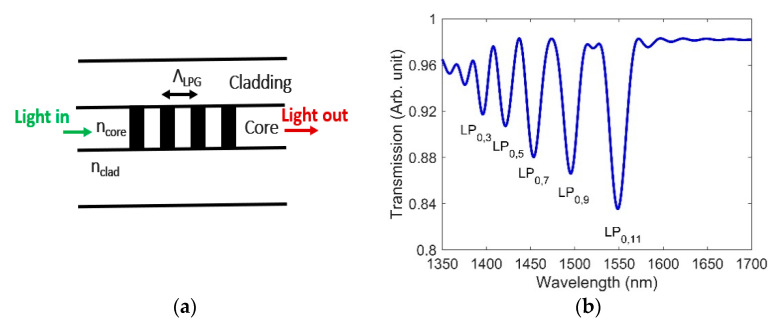
(**a**) LPG schematic, where $n_{core}$,$\;n_{clad}$, and $\Lambda_{LPG}\;$ are the RIs of the core, cladding, and grating period, respectively; (**b**) transmission spectrum of the designed LPG for $\Lambda_{LPG}$ = 348 µm and grating strength ($\Delta n)=6\times 10^{-4}$.

LPGs have also been tested in nuclear environments [12,13,28]. An LPG is similar to an FBG except that it has a larger grating period (100–1000 µm as opposed to 0.1–1 µm). In LPG, light coupling occurs between the fundamental core mode and a number of co-propagating cladding modes. Depending on the phase-matching condition, such coupling results in a number of discrete attenuation bands in the transmission spectrum [29,30]. The wavelength at which coupling takes place is called the “resonance wavelength” and is given by [30]:

(2) $$\lambda_{R}=\left(n_{eff,co}-n_{eff,cl}^{m}\right)\Lambda_{LPG}$$

where $\lambda_{R}$ is the resonance wavelength, $n_{eff,co}$ is the effective RI of the guided core mode, $n_{eff,cl}^{m}$ is the effective RI of the *m*th order cladding mode, and $\Lambda_{LPG}$ is the grating period. The resonance wavelengths are dependent on the grating period and RIs of the fiber core and cladding. The LPGs are very sensitive devices (even compared to FBG), i.e., a small change in the physical parameters can cause a large shift in the resonance wavelength. LPGs are typically used to sense temperature, pressure, and external RIs [31,32]. We chose an LPG with a grating period of 348 µm in order to achieve at least one resonance dip close to the 1550 nm wavelength, for which several sources and detectors are commercially available. The schematic and transmission spectrum of the designed LPG are shown in Figure 2a,b, respectively. For a uniform grating, the cladding modes featuring only circularly symmetric field couples to the core mode. Analysis of cladding mode resonance and coupling coefficients shows that low-order even modes (e.g., 2, 4, and 6) contain very little power in the fiber core, whereas low-order odd modes (e.g., 1, 3, and 5) have a peak localized in the core [33]. As a result, the coupling between the low-order even modes and the fundamental core mode of the fiber is expected to be very weak. In our designed LPG, the core mode couples to five odd-number cladding modes (LP_0,3_, LP_0,5_, LP_0,7_, LP_0,9_, and LP_0,11_), thus supporting the cladding mode analysis discussed in [33]. Please note that the resonance wavelength for coupling to the LP_0,11_ cladding mode is situated at 1550 nm.

### 2.3. Fabry-Perot (F-P)

F-P sensors feature several potential applications for nuclear environments, due to their insensitivity to radiation [14,15,34].

**Figure 3:**
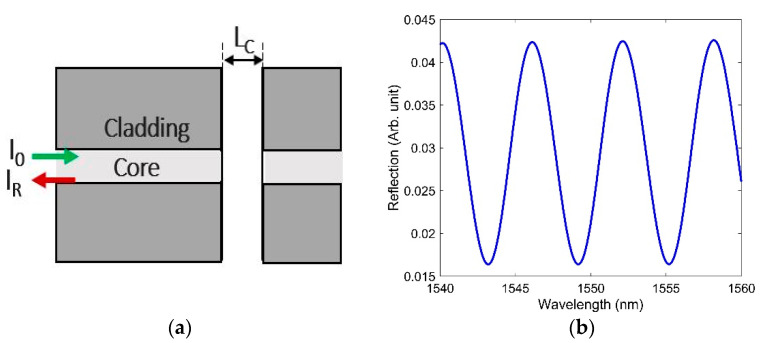
(**a**) Schematic of an EFPI, where $I_{0}$,$\;I_{R}$, and $L_{C}$ are the incoming light, reflected light, and cavity length, respectively; (**b**) interferometric fringes obtained due to interference within the cavity length $L_{C}\;$= 200 µm.

An F-P interferometer consists of two reflective surfaces surrounding a cavity. In an intrinsic F-P interferometer, the fiber, mirrors, and cavity are made within a single fiber. In contrast, in extrinsic F-P interferometer (EFPI) sensors, the cavity (containing either a different medium or air) is situated between a fiber tip and an external reflecting mirror. A schematic of an EFPI is shown in Figure 3a. The interferometric fringes of the simulated F- P sensor with a cavity length of 200 µm are shown in Figure 3b. Please note in all the designs of FBG, LPG, and F-P simulated in the paper, the same fiber parameters values as described in Section 2 were used.

## 3. Numerical Analysis of How RIC Affects OFS at Low Doses

To study how RIC affects OFS at low doses, we applied the change in RI values that resulted from the RIC observed by Kher et al. [18] (see Table 1) to our simulation models. Kher et al. derived the gamma-ray-induced RI change of a single-mode fiber by observing the resonance wavelength shift in an LPG. They used CO_2_ laser to inscribe the LPG. However, please note that inscription techniques of gratings play a crucial role in sensing different physical parameters in radiation environment. It is well known that radiation significantly affects the basic characteristics of FBG sensors such as peak wavelength and spectral width, and the amplitude and magnitude of these changes largely depend on the grating type and fabrication technique [8]. Similar to FBG, radiation also changes the structural parameters of LPG; again, it depends on the inscription technique, grating type, and composition of fibers [13,28,35]. It has been reported that fs-etched compared to conventional UV irradiated gratings can survive for a long time in radiation environment without significant degradation of gratings parameters [8,9,10]. In this section, we present our simulation results regarding how RIC affects the aforementioned sensors, based on these observed changes in the RI.

### 3.1. RIC Effects on FBG

**Figure 4:**
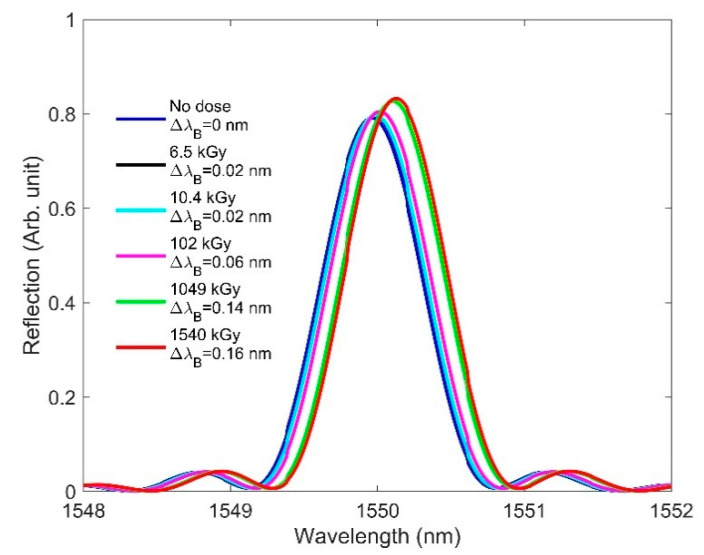
RIC effects on FBG: redshift in the Bragg wavelength from 1550 to 1550.16 nm as the dose is increased from 0 to 1540 kGy, where $\Delta \lambda_{B}$ denotes the Bragg wavelength shift.

The radiation-induced RI changes shown in Table 1 are applied in the FBG model to observe their effects on the FBG spectra. Please note that we added the RI change values to the grating portion (in both high and low RIs). Figure 4 plots the Bragg wavelength changes due to RIC at various doses. We observed that increasing the dose resulted in a redshift in the Bragg peak, since increased RI shifts the Bragg wavelength to the longer wavelength side, as per Equation (1). A high dose (1540 kGy) provides the largest Bragg peak amplitude in comparison with a no-dose condition—something unusual for an FBG under a radiation field. It was reported that increasing the radiation dose degrades the amplitude of the Bragg peak, due to the significant attenuation caused by radiation-induced color-center generation [3,6,26]. If the attenuation effects are not considered, Figure 4 would indicate that an increasing RI change resulting from the radiation dose is accompanied by an increase in the amplitude of the Bragg peak, since we are independently analyzing the RIC effect. While our preliminary simulation results showed a redshift of the Bragg wavelength as a function of increasing doses, this contradicted the experimental findings reported in [17,26,36]. In the next section, we discuss another key aspect—one that involves RIC effects and considers the length change—for accurately predicting the resonance shift behavior in FBG.

### 3.2. RIC Effects on FBG Considering Length Change

While our simulation results for FBG demonstrate a redshift of the Bragg wavelength ($\lambda_{B})\;$ due to the radiation-induced RI change, experimental results reported in [17,26,36] showed a radiation-induced blueshift of $\lambda_{B}$. Comparing the simulation results to the experimental work reported in [17,26,36] suggests that additional parameters must be considered for simulating our designs.

In our earlier simulation, we did not consider material density variations due to changes in the RI, as described by the Lorentz–Lorenz relation [16] given by:

(3) $$\frac{n^{2}-1}{n^{2}+2}=\frac{4\pi }{3}N\alpha =\frac{4\pi }{3}\left(\frac{\rho N_{A}}{M}\right)\alpha$$

where *n*,  ρ, $N_{A}$, M, and α  are the RI, density of the material, Avogadro number, molecular weight, and electronic polarizability, respectively.

A change in density modifies the volume of the fiber material and, hence, the FBG/LPG grating period as well as the cavity length for the F-P interferometer. The density change relates to the volumetric change and can be represented by the following expression:

(4) $$\frac{\rho_{2}-\rho_{1}}{\rho_{2}}=\frac{v_{1}-v_{2}}{v_{1}}=C_{v}$$

where $\rho_{1}$,$\;v_{1}$, $\rho_{2}$,$\;v_{2}$, and $C_{v}$ are the initial density, initial volume, final density, final volume, and volumetric compaction, respectively. Considering the isotropic change in volume, the relationship between volumetric compaction ($C_{v}$) and linear compaction ($C_{l}$) can be expressed by the following equation:

(5) $$C_{l}=1-{\left(1-C_{v}\right)}^{1/3}$$

A linear compaction is required to observe the effect of the length (or grating period) change caused by radiation on the resonance-based sensors. The changes in density and length due to radiation-induced RI change values were calculated using Equations (3)–(5), and the calculated results are shown in Table 2. Along with the radiation-induced RI change, we added the grating period change (linear compaction) to our FBG model in order to see their overall effect on the FBG spectra.

**Figure 5:**
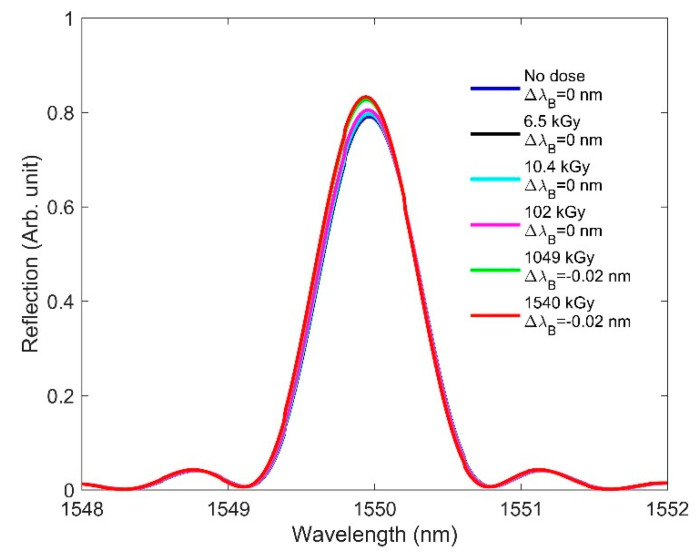
RIC effects on FBG: blueshift in the Bragg wavelength from 1550 to 1549.98 nm as the dose is increased from 0 to 1540 kGy, where $\Delta \lambda_{B}$ denotes the Bragg wavelength shift.

Figure 5 shows the Bragg wavelength shift as a function of dose when both the radiation-induced RI and length changes were considered for simulation. It is observed that, this time, the Bragg wavelength sees a blueshift with increasing doses.

While the increase in the RI produces a redshift of the Bragg wavelength, the compaction of length (reduced grating period) provides a competing blueshift. As the FBG grating period is generally very small (0.5353 µm in our design), even a small change in length caused by radiation induces a significant blueshift in the Bragg wavelength. Figure 5 reveals that the length-change effect on the Bragg wavelength compensates for the RI effect, producing a zero Bragg wavelength shift up to 102 kGy. At higher doses (1049–1540 kGy), the compaction effect on the Bragg wavelength dominates; as a result, the Bragg wavelength shifts towards the shorter wavelengths, thus supporting the experimental results reported in [17,26,36]. Please note that in [17,26,36], Nuetron-irradiation-induced shift was reported. However, both gamma and neutron irradiation alter the RI of the host fiber, although their mechanisms for changing the RI are different [37].

### 3.3. RIC Effects on LPG

**Figure 6:**
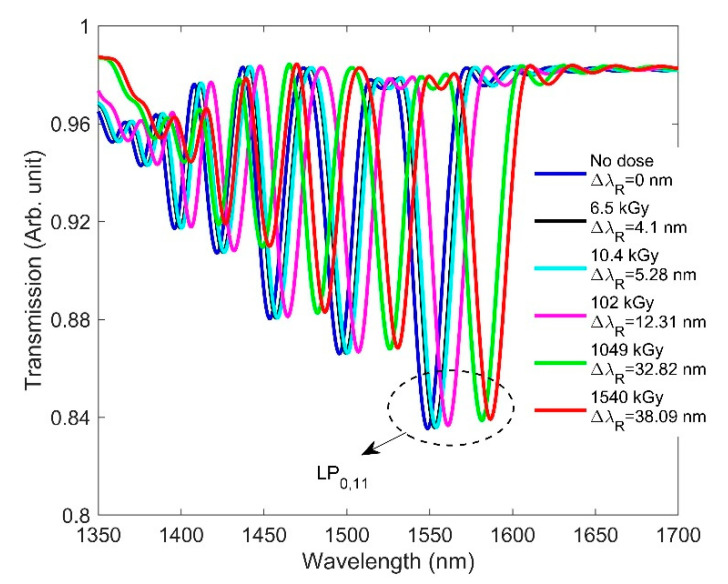
RIC effects on the transmission spectrum of the designed LPG along with the resonance wavelength shift ($\Delta \lambda_{R})$ for the LP_0,11_ cladding mode when dose is increased from 0 to 1540 kGy.

Here, we analyze how RIC affects the spectra of LPG. This is accomplished by inserting the radiation-induced RI values from Table 1 into our design. We assume no radiation-induced change in the pure silica-based cladding RI. It has been reported that pure-silica-based cladding composition has been shown to be resistant to gamma up-to 1 MGy due to the absence of precursors of radiation induced color centers like NBOHC and E’-centers [38]. Since no radiation-induced change in the cladding RI is considered, the resonance wavelength is expected to shift significantly due to the large effective RI difference between the core and cladding ($n_{eff,co}-n_{eff,cl}^{m}$). As with FBG, the RI change values due to radiation were added to the grating portion. Figure 6 shows the transmission spectrum for the designed LPG when only the RI change due to radiation was considered in the simulation. Please note that, throughout the paper, we only considered the resonance wavelength shift of the LP_0,11_ cladding mode. The resonance wavelength is seen to shift from 1550 to 1588.08 nm when the accumulated dose is increased from a no-dose condition to 1540 kGy. This is due to the dependency of the resonance wavelength on the differential RI between the core and cladding. The simulation results show good agreement with the experimental results reported in [18], where the researchers demonstrated a redshift of the resonance wavelength with increasing doses.

### 3.4. RIC Effects on LPG Considering Length Change

**Figure 7:**
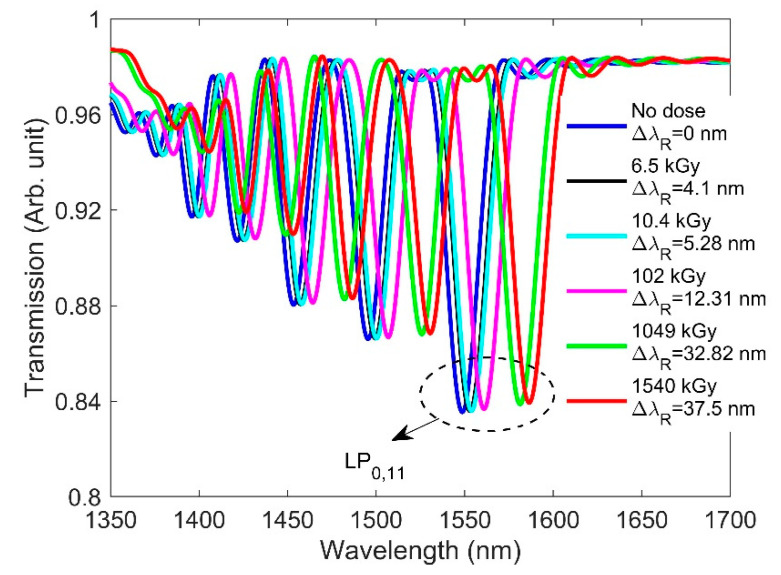
RIC effects on the transmission spectrum of the designed LPG along with the resonance wavelength shift ($\Delta \lambda_{R})$ for the LP_0,11_ cladding mode when the dose is increased from 0 to 1540 kGy.

Figure 7 shows the combined effect of the radiation-caused RI and length changes on the spectra of the designed LPG. It is seen from Figure 7 that the resonance wavelength shift is slightly smaller than when the length change was not considered (Figure 6), due to the competing blueshift effect induced by the length change effect. Since the grating period of the designed LPG is 348 µm, a small change in the grating period due to the radiation does not significantly affect the resonance wavelength. Furthermore, the resonance wavelength shift of LPG under gamma radiation was observed, which cannot be attributed to changes in grating period [18]. As a result, the effect of the RI on the LPG spectra dominates over the length compaction effect, and the resonance wavelength shifts towards the longer wavelength for increasing doses.

### 3.5. RIC Effects on F-P

**Figure 8:**
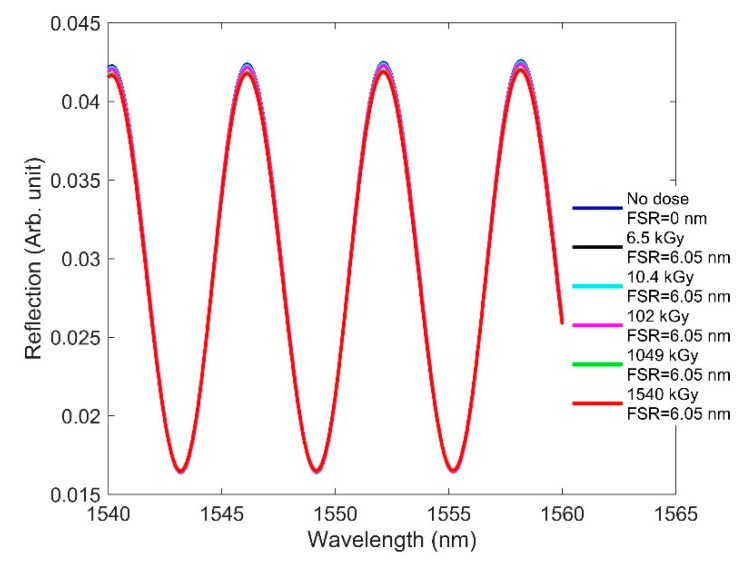
RIC effects on the interferometric fringes of the designed F-P when the dose is increased from 0 to 1540 kGy, where FSR is the spectral distance between two adjacent fringe peaks for a given radiation.

Here, we focus on how RIC affects F-P sensor performance. We designed an EFPI in which the cavity is comprised of an air medium. We considered one end surface of the cavity to be metal and the other to be the fiber facet. Because of the air cavity, the radiation-induced RI change to the fiber did not affect the wavelength characteristics of the fringes. As a result, the free spectral range (FSR)—namely, the spectral distance between two adjacent fringe peaks—remained constant with increasing doses (Figure 8).

### 3.6. RIC Effects on F-P Considering Length Change

**Figure 9:**
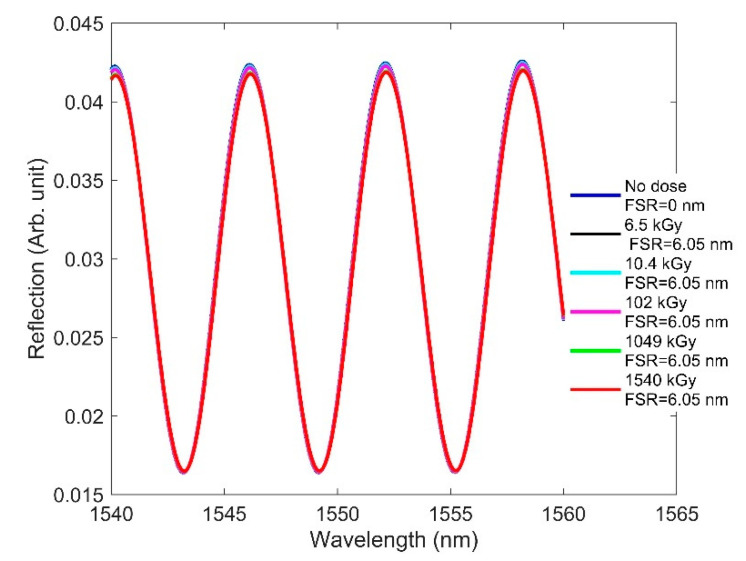
RIC effects on the interferometric fringes of the designed F-P when the dose is increased from 0 to 1540 kGy, where FSR is the spectral distance between two adjacent fringe peaks for a given radiation.

It is necessary to consider the radiation-induced length change to observe how RIC affects the designed F-P sensor, even though the cavity medium is air. For simulation purposes, we considered a roughly 50-µm-long fiber segment exposed to radiation beyond the fixing point, for example, as in [15], and compaction was calculated for that length. The responses of the sensor can differ, depending on the choice of cavity length as well as the fiber length exposed to a radiation field. For example, F-P sensors with different lengths of lead-in fibers were intentionally exposed to radiation, and compaction was assessed on the fiber tips in [15]. The calculated length change taken from Table 2 was used to simulate the F-P sensor. Figure 9 shows the fringe pattern and FSR of the designed F-P sensor at different doses. We observed no change in the fringe position, owing to a similarly negligible change in the effective cavity length. Of the three types of sensors studied in regard to low-dose effects, the F-P sensor was found to be the most robust to radiation. Thus, F-P sensors can be considered a potential candidate for measuring physical parameters in nuclear environments when the accumulated dose reaches 1540 kGy.

## 4. RIC Effects on OFS at High Doses

**Figure 10:**
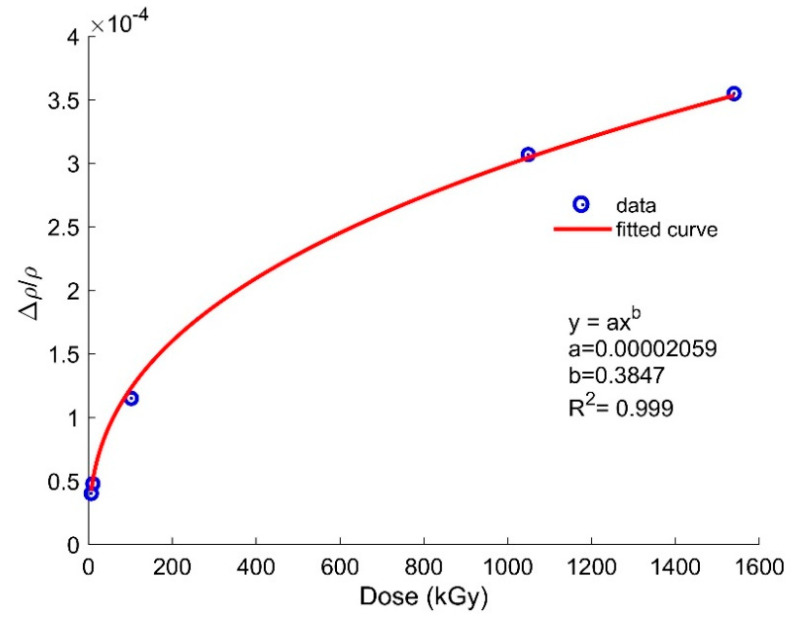
Curve fitting for relative density as a function of dose using the values from Table 2.

**Figure 11:**
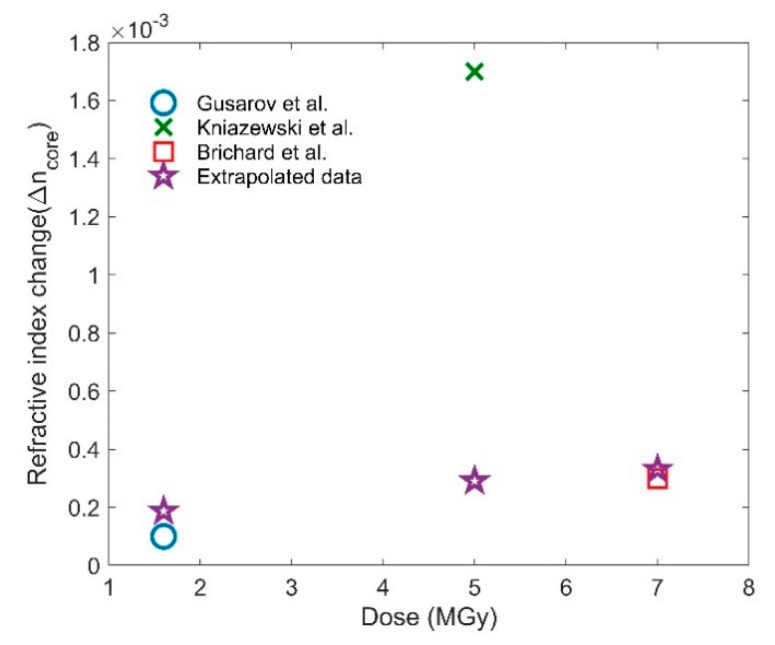
Calculated RI with the experimental values at MGy dose.

As discussed earlier, radiation alters the RI by means of compactions and creating diferetnt types of defects in the host fiber. Several works have observed the gamma-radiation-induced change in RI values in MGy doses [39,40,41], which are shown in Table 3. It is seen that the change in RI is not consistent with the accumulated doses since several factors, such as dose rate, composition of fibers, and irradiation temperature, can affect the RI change. So far, we have considered radiation effects at lower doses. To study the effects on OFS at high doses, we used the radiation-induced density values from Table 2 and fitted the relative density vs. dose plot using a power law fit (see Figure 10). Using the fitting constant values, we calculated the density, RI, and length changes for the sensors at higher doses of up to 7 MGy via the following power law equation [37]:

(6) $$\frac{\Delta \rho }{\rho_{1}}=AD^{C}$$

where *A* is the material-dependent constant and *C* is another constant [37]. Based on fitting parameters, we determined the values of *A* and *C* to be 0.00002059 and 0.3847, respectively. By inserting the values of the fitting constants into Equation (6), the RI and length compaction were calculated for doses of 1.6, 5, and 7 MGy. The extrapolated data calculated by our approach is shown in Table 4. To validate our extrapolation, we found three experimental works within MGy regime with their modified change in RI [37,38,39] within the core shown in Figure 11 along with our extrapolated data (star marker). The details of the three experimental works are given in Table 3.

It can be seen that our extrapolated data is consistent with the RI change reported by Gusarov et al. [37] and Brichard et al. [38], while the data provided by Kniazewski et al. [39] for 5 MGy is within the order of magnitude of our prediction. We limit our work to MGy as we only found experimental validations within MGy levels. Please note that there are different factors that alter the optical and mechanical properties of the fiber exposed to gamma radiation, which has been discussed in the Section 1. In the next subsections, we analyzed the effects of both RI and length change at these MGy doses for the three sensor types.

### 4.1. RIC Effects on FBG

**Figure 12:**
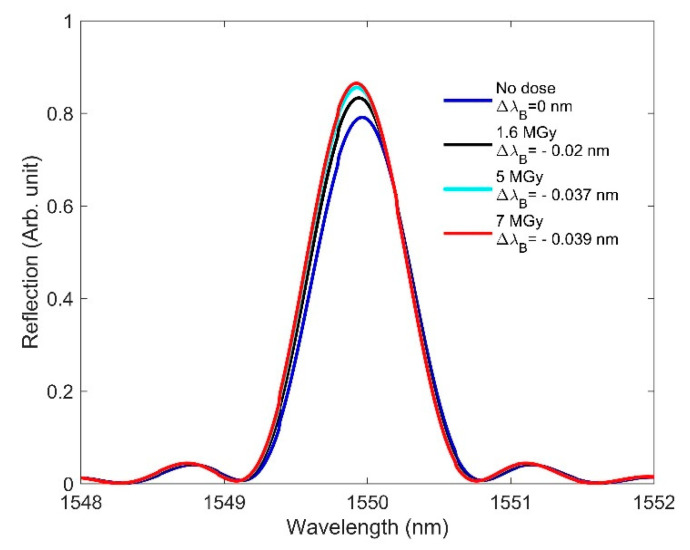
RIC effects on FBG at high doses: blueshift of 0.039 nm in the Bragg wavelength when the dose is increased from 0 to 7 MGy and radiation-induced changes in length and RI are considered.

The effects of RIC on the FBG spectra at high doses, which are similar to those observed at low doses, are shown in Figure 12, wherein, as is consistent with earlier observation, we see a significant blueshift in the Bragg wavelength when increasing the dose from 0 to 7 MGy. As the radiation-induced changes in length compaction and RI are significant at high doses, the Bragg wavelength shows a large blueshift of 0.039 nm. At high doses, the length compaction effects on the Bragg wavelength completely dominate the RI effect by producing a blueshift.

### 4.2. RIC Effects on LPG

**Figure 13:**
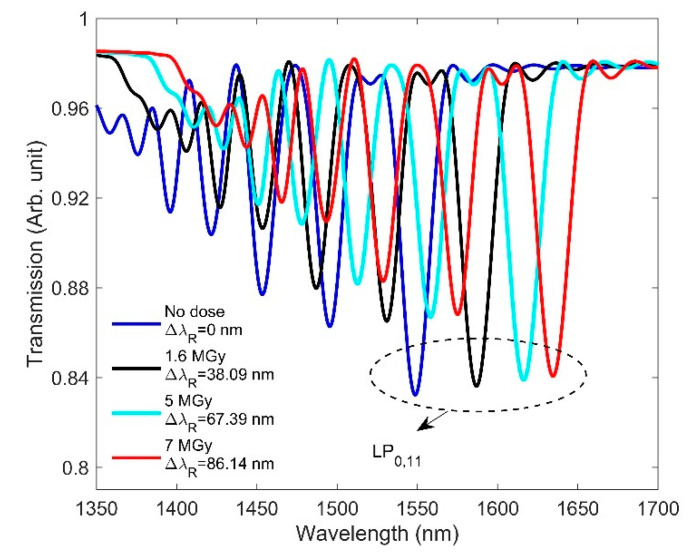
RIC effects on the transmission spectrum of the designed LPG at high doses.

The effect of RIC on LPG at high doses is shown in Figure 13. It is seen that, as the dose increases from 0 to 7 MGy, the resonance wavelength shifts significantly towards longer wavelengths. As mentioned in Section 3.2, a small radiation-induced RI change creates a large effective RI difference ($n_{eff,co}-n_{eff,cl}^{m}$) between the core and cladding modes. Table 4 shows that the radiation-induced RI change is large enough to produce a 86.14 nm redshift of the resonance wavelength at a dose of 7 MGy. Although the radiation-induced length compaction is large at high doses, it has little effect on the resonance wavelength shift. This is because, compared to the grating period of the designed LPG, it is still very small. For a better understanding of how radiation affects LPG, it is important to consider the RI change in the coupled cladding modes from the phase matching curve in order to correct the excessive shift of the resonance wavelength [42].

### 4.3. RIC Effects on F-P

**Figure 14:**
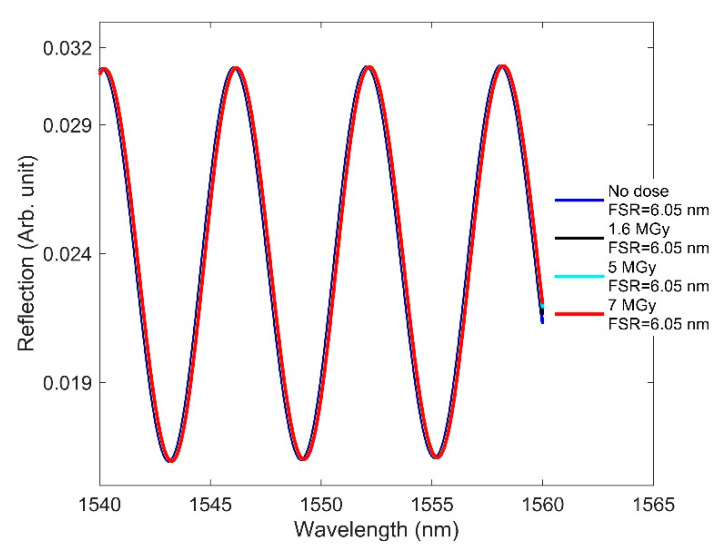
RIC effects on the interferometric fringes of the designed F-P sensor at high doses.

The effect of RIC on the spectrum of the F-P interferometer at high doses is shown in Figure 14. Even at high doses, no change of fringe spacing can be observed. As the exposed length utilized to observe the compaction effect was only 50 µm in our designed F-P sensor, the radiation-induced length compaction remains very small compared to the cavity length of 200 µm. The cavity length will change significantly—and hence the FSR as well—if the length exposed to the radiation is large and the compaction is assessed using that large length.

### 4.4. RIC-Induced Temperature Error

The temperature sensitivities of SMF-28-based FBG and LPG sensors are 10 and 92 pm/°C, respectively [9,16,29,31]. For F-P sensors, depending on their structure, the sensitivity can differ: 13.6 pm/°C [43], 1.56 and 1.87 nm/°C [44], 5.2 nm/°C [45], and 11.86 and 19.55 nm/°C [46]. Radiation-induced shift in wavelength of fiber sensors causes temperature error if these sensors are used for temperature sensing. Table 5 shows the temperature errors of these three types of sensors at different doses. Please note that the temperature error for the F-P sensor was calculated by considering the lowest sensitivity of 13.6 pm/°C. We observe that, in LPG, a small change in core RI due to radiation induces a significant wavelength shift and hence the radiation-induced temperature error, most probably as a result of not considering the radiation-induced RI change of the coupled cladding modes. For FBG and F-P, even at high doses the radiation-induced temperature error is quite small, as is acceptable in nuclear environments. We calculated errors based on the wavelength shift for all sensors. While the maximum error in temperature measurement for FBG is 3.9 °C, it is only 0 °C for F-P. Based on the wavelength shifts of the three types of sensors, the F-P sensor seems to show the most radiation-resistant response under high-radiation environments, both at low and high doses.

## 5. RIA Effects on OFSs

In this section, we present the independent effect of RIA on the three types of OFS. RIA darkens fibers under radiation environments, and is heavily dependent upon dopants and defects [3,16,17]. Each different fiber composition will produce a different RIA effect. However, the overall trends remain consistent for any type of fiber. The RIA values obtained in [47,48] are shown in Table 6, and these were used when designing sensors in FIMMWAVE. To compute the effect of RIA, we inserted into our sample FBG, LPG, and F-P models (discussed in Section 2) the loss values from Table 6, as obtained from [47,48]. To observe how RIA affects FBG and F-P, we assumed that a 5-m length of fiber was exposed to radiation.

**Figure 15:**
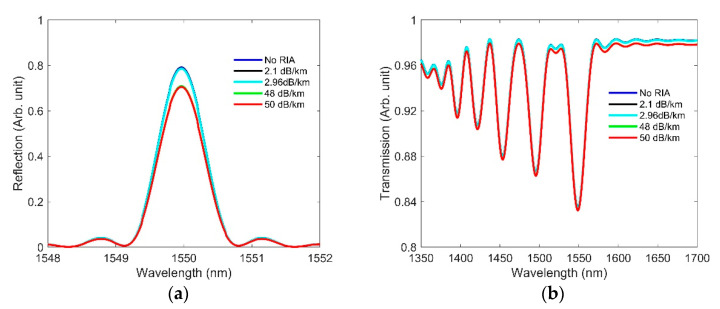
Effect of RIA on the spectra of (**a**) FBG and (**b**) LPG, using the same design parameters applied earlier.

**Figure 16:**
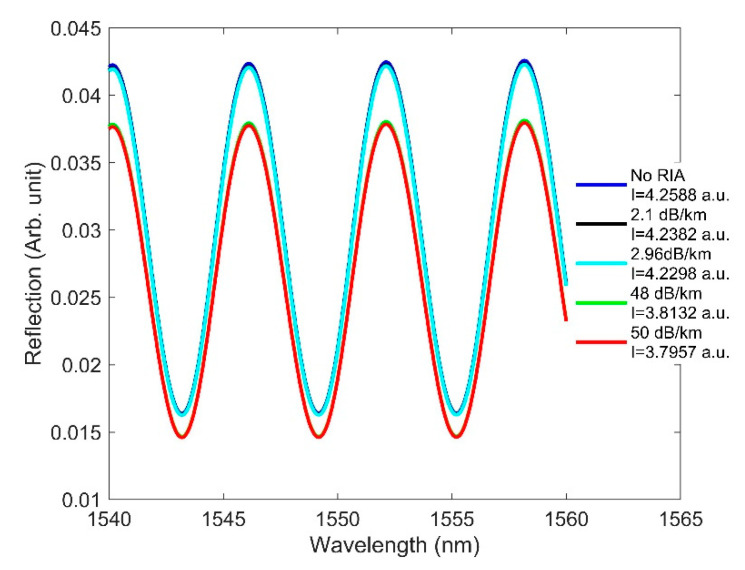
Effect of RIA on F-P at various doses, using the same design parameters applied earlier.

The intensity was then calculated using different radiation values for these sensors. The RIA effects on the three types of sensors are shown in Figure 15 and Figure 16. The amplitude of the reflected light in FBG (Figure 15a) and F-P (Figure 16) is seen to reduce with increasing RIA, as expected. The LPG sensor responded to increasing RIA with a larger dip in its transmitted spectrum (improved contrast), whereas no change in contrast occurred for FBG and F-P as a result of RIA. We believe this is because the sensor lengths for FBG and F-P are very small compared to that of LPG. In the case of FBG, there was no contrast variation, since RIA affects the Bragg and sidelobe peaks equally. It is also apparent from Figure 15 and Figure 16 that RIA only reduces the signal strength, without affecting the resonance wavelength position. Future work is needed to include the effects of both RIC and RIA and compare them against experimental results.

## 6. Conclusions

The RIC effects on the three types of OFS have been explored at both low and high doses. We modeled the base design for the three types of OFS and plotted their corresponding spectra under no-dose conditions. Next, we showed numerically how a radiation-induced change in RI and length affect the spectra of sensors at doses of up to 1540 kGy. Upon observation of wavelength shift due to radiation, FBG, LPG, and F-P showed a wavelength shift of 0.02, 37.5, and 0 nm at a dose of 1540 kGy, which corresponded to a temperature errors of 2°, 407°, and 0 °C, respectively. The temperature errors indicated that the F-P sensor has a radiation-insensitive response among the three discussed resonance-based sensors. Next, we calculated the change in density, RI, and linear compaction at high doses based on the values at low doses using power law equation. Then, the calculated values were used in our model to study the effects of RIC at up to 7 MGy. Even at high doses, the F-P sensor showed no shift of wavelength and hence no error in temperature measurement, making it the best candidate for a nuclear environment, followed by FBG and then LPG.

## Figures and Tables

**Table 1:** Radiation-induced RI changes in a B/Ge codoped fiber reported by Kher et al. Adapted with permission from ref. [18]. Copyright 2013 IEEE.

Dose (kGy)	$\mathrm{Refractive}\;\mathrm{Index}\;\mathrm{Change}\;(\Delta n_{core})$
6.5	$0.21\times 10^{-4}$
10.4	$0.25\times 10^{-4}$
102	$0.60\times 10^{-4}$
1049	$1.60\times 10^{-4}$
1540	$1.85\times 10^{-4}$

**Table 2:** Calculated values of density and length compaction (based on the RI values) using Equations (3)–(5).

Dose (kGy)	$\Delta n_{core}\;$	$\frac{\Delta \rho }{\rho_{1}}\left(\%\right)\;$	$C_{v}(\%)$	$C_{l}(\%)$
0	0	0	0	0
6.5	$0.21\times 10^{-4}$	0.00403	0.00403	0.00134
10.4	$0.25\times 10^{-4}$	0.00479	0.00479	0.00159
102	$0.60\times 10^{-4}$	0.01152	0.01152	0.00384
1049	$1.60\times 10^{-4}$	0.03071	0.03069	0.01023
1540	$1.85\times 10^{-4}$	0.03551	0.03549	0.01183

**Table 3:** Gamma-radiation-induced RI change in different compositional fibers at MGy dose.

Type of Fiber	Dose Rate	Total Dose	$\Delta n_{core}\;$	Refs.
Telecom grade	N/A	1.6 MGy	$\sim 10^{-4}$	Gusarov et al. [39]
SMF-28	23 kGy/h	5 MGy	$1.7\times 10^{-3}$	Kniazewski et al. [41]
Ge, N doped	20 kGy/h	7 MGy	$3\times 10^{-4}$	Brichard et al. [40]

**Table 4:** Calculated values for higher doses, as obtained by inputting the fitting constant values from Figure 10 into Equation (6).

Dose	$\Delta n_{core}\;$	$\frac{\Delta \rho }{\rho_{1}}\left(\%\right)\;$	$C_{v}(\%)$	$C_{l}(\%)$
1.6 MGy	$1.87\times 10^{-4}$	0.03589	0.03587	0.01196
5 MGy	$2.91\times 10^{-4}$	0.05580	0.05577	0.01859
7 MGy	$3.31\times 10^{-4}$	0.06357	0.06353	0.02118

**Table 5:** Radiation-induced temperature errors in FBG, LPG, and F-P sensors at higher doses.

Dose	Temperature Error		
	FBG	LPG	F-P
1.6 MGy	2 °C	414 °C	0
5 MGy	3.7 °C	732 °C	0
7 MGy	3.9 °C	936 °C	0

**Table 6:** RIA values obtained from [40,41].

Dose (kGy)	Dose Rate (rad/min)	Loss (dB/km)	Loss (cm^−1^)
0	0	0	0
1	0.01	2.1	0.00009
1	0.1	2.96	0.00012
66	333	48	0.00208
85	167	50	0.00217

## Data Availability

Not applicable.
